# Size-Dependent Ion Adsorption in Graphene Oxide Membranes

**DOI:** 10.3390/nano11071676

**Published:** 2021-06-25

**Authors:** Xiaoheng Jin, Xinyue Wen, Sean Lim, Rakesh Joshi

**Affiliations:** 1 School of Materials Science and Engineering, University of New South Wales (UNSW), Sydney, NSW 2052, Australia; xinyue.wen@unsw.edu.au; 2 Electron Microscopy Unit, Mark Wainwright Analytical Center, University of New South Wales (UNSW), Sydney, NSW 2052, Australia; sean.lim@unsw.edu.au

**Keywords:** adsorption, graphene oxide, size effect

## Abstract

Graphene oxide (GO)-based materials have demonstrated promising potential for adsorption and purification applications. Due to its amphiphilic nature, GO offers the possibility of removing various kinds of contaminants, including heavy metal ions and organic pollutants from aqueous environments. Here, we present size-selective ion adsorption in GO-based laminates by directly measuring the weight uptake of slats. Adsorption studies were conducted in graphene oxide purchased from Nisina Materials Japan prepared using a controlled method. We tuned the interlayer spacing of GO membranes via cationic control solutions using intercalation of very small salts ions (i.e., K^+^, Na^+^, Cl^−^) very precisely to facilitate the adsorption of larger ions such as [Fe(CN)_6_]^4−^ and [Fe(CN)_6_]^3−^. This study demonstrates that if the opening of nanocapillaries within the laminates is bigger than the hydrated diameter of ions, the adsorption occurs within the membranes while for smaller opening, with no ion entrance the sorption occurs on the surface of the membranes.

## 1. Introduction

As an emerging solution-processable class of nanomaterials, graphene oxide (GO) has shown enormous potential for use in molecular sieving [1,2], ion separations [3,4,5], desalination [6,7,8,9], purification [10,11,12], supercapacitors [13], electronics [14,15], and lithium ion-based batteries [16]. By stacking the GO sheets layer by layer, the assembled membrane has a laminated structure where the interlayer spacing determines mass transport behaviour. Pristine GO membrane shows a strict rejection of ions and molecules with a hydrated radius larger than 4.75 Å [17]. To this end, the excellence of GO membranes for practical applications primarily relies on the control of interlayer spacings which indeed, has been a subject of interest for many studies [1,4,18,19,20,21,22,23]. However, due to the hydrophilic nature of graphene oxide [24], the laminates swell when hydrated, expanding the interlayer spacing to more than one nanometer [25]. Efforts have been made to avoid the swelling of graphene oxide laminates. For example, Nair et al. control the interlayer spacing of GO membranes between epoxy for desalination purposes [26]. Further, when GO membranes are intercalated with cations, the negatively charged oxygen functional groups can be interlinked by the ions to prevent the swelling of the laminates [2].

Given the electrostatic interaction with cations, graphene oxide has been studied extensively as a heavy metal ion adsorbent in an aqueous environment. The electrostatic force makes negatively charged graphene oxide flakes attractive to heavy metal cations, and thus provides excellent sorption performance in aqueous solutions [2]. Indeed, graphene oxide-based adsorbents have been reported to effectively remove ions such as palladium, gold, mercury, and lead [2,27,28,29]. It is also shown that GO-based adsorbents share excellent reproducibility regardless of the assembly method [2]. Apart from removing cations by physicochemical adsorption, graphene oxide can also act as a surfactant to remove organic molecules with larger molecular radius, such as emerging organic contaminants (EOCs) [30] and polycyclic aromatic hydrocarbons (PAHs) [31] from aqueous sources. GO-based adsorbent can soak hydrophilic as well as hydrophobic contaminants from water because the hydrophobic graphitic area and hydrophilic functional groups make each GO flake an individual surfactant sheet [32]. 

**Figure 1:**
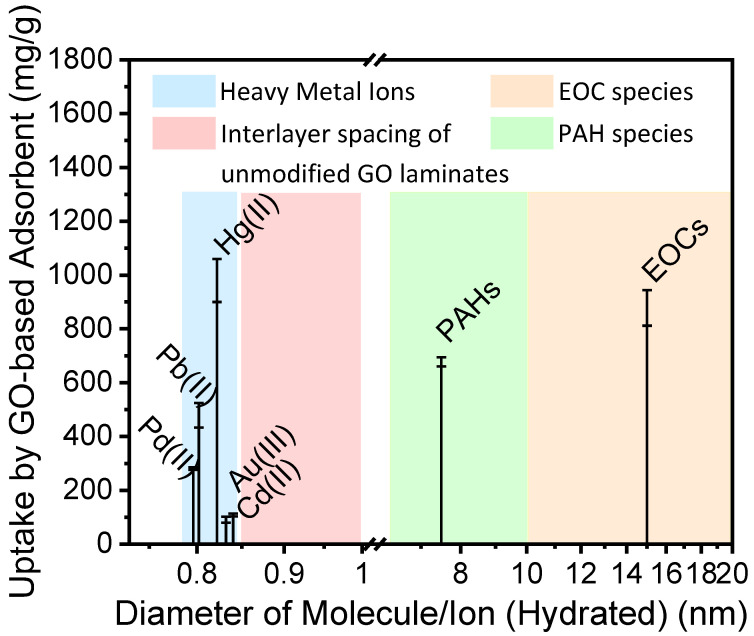
Adsorption amount of ions and molecules by GO-based adsorbent, including heavy metal ions [27,28,29,37], Emerging organic contaminants (EOCs) [30] and polycyclic aromatic hydrocarbons (PAHs) [31].

Figure 1 summarizes the radius of ions and molecules identified as major contaminants and their uptake amount by GO-based adsorbents. As Figure 1 demonstrates, very few studies have focused on adsorption of ions and molecules with a hydrated diameter between 8.5 to 10 Å, using GO-based adsorbents. Depending on the preparation method, the interlayer spacing of GO laminates varies from 8.5 to 10 Å [33,34,35]. The GO-based adsorbents have not been used significantly in the past due to the possible variation of interlayer spacing of GO in the presence of water [25,36]. As a result, it is difficult to obtain reliable adsorption amount from the contaminants with a radius in this range. However, artificial system with size-dependent separation function is of great interest for modern separation technology such as the extraction of Li^+^ from seawater and removal of ions such as Cs^+^ from radioactive waste. 

This work is the first attempt to systematically study the secondary adsorption of ions close to the interlayer spacing of a graphene-based laminated adsorbent. A wide variety of parameters on sorption behaviour has been studied, including temperature, concentration, oxygen functional groups and sorption time. We used cationic control to regulate the interlayer spacing of GO laminates for studying its adsorption properties to ions with a diameter between 8 to 10 Å. We have selected especially the two ions [Fe(CN)_6_]^4−^ and [Fe(CN)_6_]^3−^. Here, the adsorbates (Fe (II) and Fe (III)) were chosen based on two considerations: (1) similar molecular structure so that the chemical sorption component can be neglected; and (2) slightly different hydrated diameter (8.44 Å for [Fe(CN)_6_]^4−^ and 9.50 Å for [Fe(CN)_6_]^3−^, [38]) which is close to the reported diameter cut-off for GO membrane filtration [17]. Graphene oxide membranes were prepared by vacuum filtration, as this method provides appropriate simplicity and stable laminated structure [39].

## 2. Materials and Methods 

### 2.1. Preparation of GO Membranes 

The GO nanosheets (size < 500 nm) used in this study prepared *via* modified Hummer’s method (further modified) were supplied by Nisina Materials (Okayama-city, Japan) and to exfoliate, the solution was further ultrasonicated, followed by centrifugation to separate the layers. The obtained suspension was washed following the reported process [40] to exclude the remaining ions. The graphene oxide suspension was then filtered through a polyvinylidene fluoride (PVDF) substrate to obtain graphene oxide membranes. The as-prepared GO membranes were stored in desiccators before analysis. The size of nanoflakes as well as the charge in membranes may have important role in adsorption and diffusion. Small flake size of GO or large flake- to- flake lateral distance may lead to higher effective porosity in membranes. 

### 2.2. Interlayer Spacing Control of GO Membranes and Swelling Test

It is known that the interlayer spacing of GO membranes varies when it is soaked in different salt solutions [2]. To ensure the reliability of our experiment, all GO membranes used in this study were taken from the same stock solution. The GO membranes were soaked with salt solutions (KCl, NaCl, CaCl_2_, and MgCl_2_) at controlled concentration and temperature for two hours. After the sorption, the membranes were carefully transferred into dry centrifuge tubes. The remaining solution was removed by centrifuging the membrane at 3000 rpm for 10 min. X-ray diffraction was utilized to monitor the interlayer spacing change before and after the interlayer spacing control process. For studying the swelling behavior, the GO membranes with cationic control were washed by deionized water, and test again by X-ray Diffraction (XRD) analysis. This method of determining swelling behaviour was described in detail in literature [41].

### 2.3. Salt Adsorption under Interlayer Spacing Control 

To obtain the adsorption amount of salt in GO membranes with cationic control, we adopted a method reported in the literature [2]. Briefly, the membranes were immersed in cationic control solutions for 1 h, analyzed by XRD, and then immersed in cyanide solution for 1 h, and again analyzed by XRD. This method reported here is based on two assumptions: (1) volume change of adsorbate solution caused by GO membrane is negligible; (2) adsorption of cation and anion on GO membrane is nearly stoichiometric so that the amount of salt being adsorbed relates to the number of ions being adsorbed. To calculate the salt uptake, GO membranes with known weight (M) were firstly immersed in 0.25 mol/L of chloride salts (KCl, NaCl, CaCl_2_, and MgCl_2_), and then moved to adsorbate solutions of K_4_[Fe(CN)_6_] and K_3_[Fe(CN)_6_] with a concentration of 0.1 M, 0.5 M and 0.7 M with a volume V. The samples were stored at different temperatures for at least two hours until the equilibrium was considered as reached. In the tested temperature range, the swelling of GO membrane is considered minimal. The concentration of adsorbate before and after adsorption (*C_B_* and *C_A_*) was measured by inductively coupled plasma optical emission spectrometry (ICP-OES). The weight uptake of adsorbates (*Q_uptake_* (*T*)) can be expressed by:

(1) $$Q_{uptake}\left(T\right)=\frac{\left(C_{B}-C_{A}\right)V}{M},$$

The temperature of the solution was maintained by a water bath, with a standard error of ±0.1 °C. The concentration of the ions of interest was obtained by inductively coupled plasma optical emission spectrometry (systemic error 10^−5^ mol/L), where the Fe was selected as a benchmark element for all related ions. The weight of the membranes was recorded by a high precision electrical balance, with a measured standard error of 0.01%. 

### 2.4. Characterizations 

Morphology observation of the membranes was carried out by scanning electron microscopy (SEM, NanoSEM 450, FEI Nova, Hillsboro, OR, USA), and transmission electron microscopy (TEM, cm200, Philips, Amsterdam, The Netherlands). A cross-sectional TEM specimen was prepared by focused ion beam (FIB, FEI Nova Nanolab 200, Hillsboro, OR, USA). The X-ray diffraction (XRD) signals were collected from an Empyrean I system (Malvern PANalytical, Westborough, MA, USA). To check if there is residual salt, all XRD signals weres recorded up to 50 degrees. Measurement of the solution-soaked membrane with XRD was carried out on membranes sealed with polycarbonate bags to avoid evaporation. The X-ray photoemission spectroscopy data was collected from a Thermo Fisher ESCALAB250Xi instrument (Thermo Fisher, Waltham, MA, USA). The determination of concentration was carried out on an inductively coupled plasma optical emission spectrometer (ICP-OES, Optima, Perkin Elmer, Waltham, MA, USA). The specific surface area of the membrane was calculated from the nitrogen physisorption isotherms of 20 milligrams of the sample at 77 K, which was obtained on a Tristar 3000 device (Micromeritics, Cumming, GA, USA).

## 3. Results and Discussion

**Figure 2:**
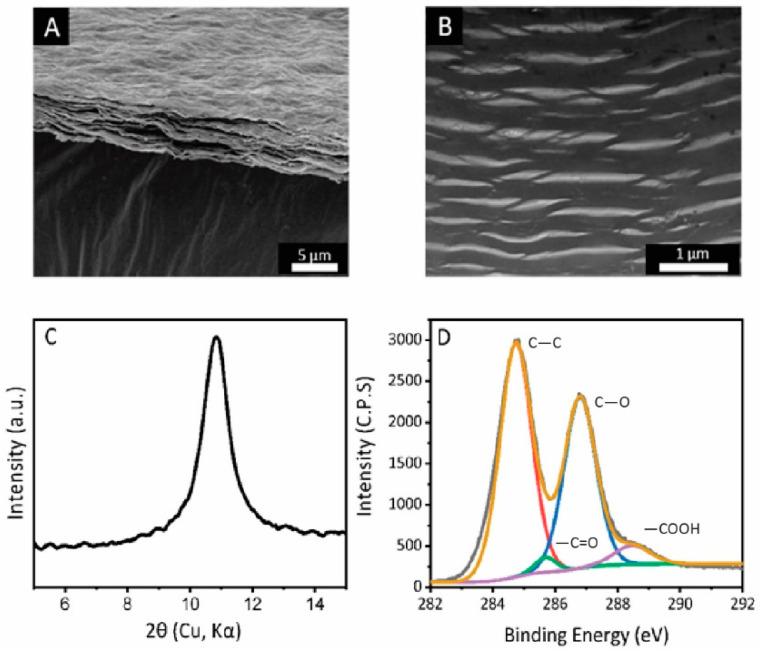
(**A**) SEM image of as-prepared graphene oxide membrane. Scale bar: 5 μm. (**B**) Cross-sectional view of as-prepared graphene oxide membrane by FIB-TEM. Scale bar: 1 μm. (**C**) XRD spectra of as-prepared graphene oxide membrane. (**D**) XPS spectra of as-prepared graphene oxide membrane, with a labelled composition of carboxyl, hydroxyl, and carbon to carbon bond.

The GO membranes prepared by the vacuum filtration share a similar thickness of 2 μm, and a similarly wrinkled membrane surface (Figure 2A). The specific surface area of the membranes is 45 ± 5 m^2^/g, which origininates from to the porous structure as observed by cross-sectional FIB-TEM (Figure 2B). The average interlayer spacing (using XRD analysis) of GO laminates was observed to be 7.8 ± 0.2 Å, which is typical for our membranes (Figure 2C). The carbon to oxygen ratio for the membranes is 2.40 ± 0.05, with an identical distribution of carboxyl, hydroxyl and ester groups (Figure 2D). 

Our experiments show that GO membranes, when immersed in deionized water without external pressure, swell by water molecules and thus enlarges its interlayer spacing to more than 1 nm. As the thickness of monolayer graphene is 3.4 Å [42], it is essential to precisely confine the interlayer spacing of GO membranes between 11.4 to 13.4 Å to study the adsorption of ions with a diameter between 8 to 10 Å.

### 3.1. Cationic Control of the Interlayer Spacing of GO Membranes

**Figure 3:**
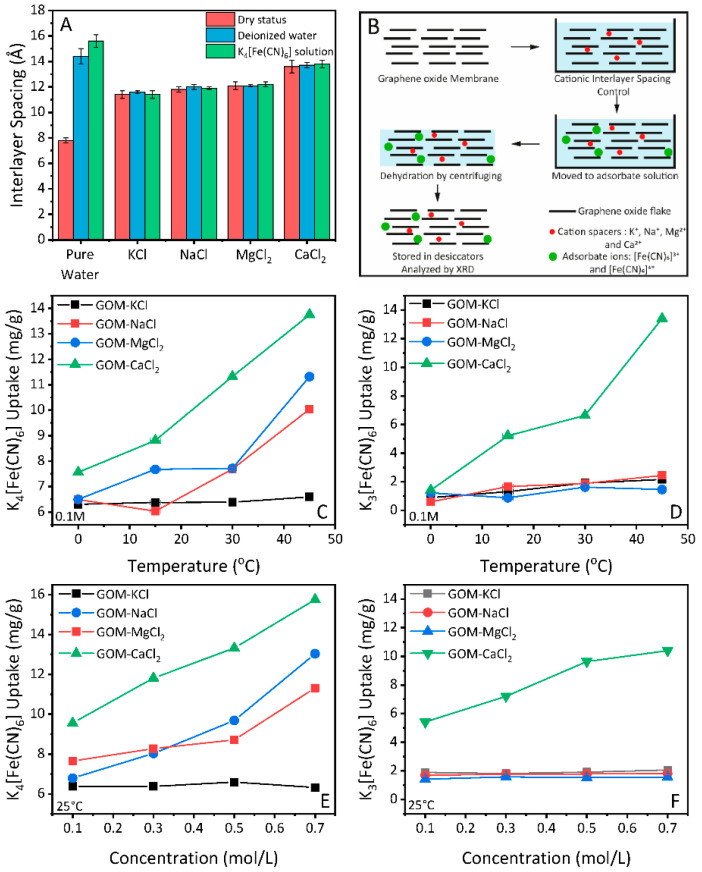
(**A**) Interlayer spacing of GO membranes with cationic control, and the swelling behaviour of GO membranes immersed in deionized water and K_4_[Fe(CN)_6_] solution. (**B**) Schematic of GO membranes with cationic control and adsorption uptake analysis. (**C**,**D**) Salt uptake by GO membrane with cationic control for K_4_[Fe(CN)_6_] and K_3_[Fe(CN)_6_] with temperature. (**E**,**F**) Salt uptake by GO membrane with cationic control for K_4_[Fe(CN)_6_] and K_3_[Fe(CN)_6_] with concentration.

To minimize swelling during the adsorption process of GO membranes, we used cationic control method [2]. Graphene oxide membranes were firstly dipped in cationic control solutions, then moved to deionized water for the swelling test. Specifically, the interlayer spacing of GO membranes controlled by KCl exhibit an average value of 11.4 Å, while intercalation of NaCl, CaCl_2_, and MgCl_2_ further enlarges the interlayer spacing of GO membranes to 11.8 Å, 12.1 Å and 13.6 Å, respectively (Figure 3A, red columns). The value of interlayer spacing for cationic controlled GO membranes was averaged from multiple measurements on three different membranes. Here, we use the term such as KCl-GO to represent the GO membranes with cationic controlled interlayer spacing. 

Evaluation of the swelling behaviour was conducted in the XRD chamber, with a method described elsewhere in detail [41]. Briefly, GO membranes were immersed in deionized water, then scanned by XRD. Opposite to the unmodified ones, the GO membranes with cationic control exhibit resistance to swelling in deionized water (Figure 3A, blue columns). It has been reported that GO membranes with cationic control by Ca^2+^, Mg^2+^ and Mn^2+^ can keep their structural integrity in deionized water for over one month [2,40], significantly longer than the time required for adsorbates to reach equilibrium inside/outside the membrane. 

### 3.2. Adsorption Properties of GO Membranes under Cationic Control

We then evaluated the adsorption properties of GO membranes toward the [Fe(CN)_6_]^4−^ and [Fe(CN)_6_]^3−^ in the membranes as a function of temperature. Prior to the adsorption analysis, it is necessary (as discussed in the introduction.) to check the swelling properties of GO membranes with cationic control in adsorbate solutions. Therefore, the as-prepared GO membranes were soaked with cationic control solutions (KCl, NaCl, MgCl_2_ and CaCl_2_) respectively, and then immersed in 0.1M of K_4_[Fe(CN)_6_] solution. The membrane (immersed) was analyzed by XRD to check the swelling properties. The schematic for the adsorption experiment under cationic control is shown in Figure 3B. We found that the GO membranes with cationic control were comparably stable in the adsorbate solution, as shown in Figure 3A (green columns). Based on these results, we attempted to calculate the amount of adsorbed K_4_[Fe(CN)_6_] and K_3_[Fe(CN)_6_] as described in the experimental section.

Figure 3C,D present how the temperature affects the weight uptakes of those cationic controlled GOMs upon being placed in the 0.1M K_4_[Fe(CN)_6_] and K_3_[Fe(CN)_6_] solutions. For the smaller ion ([Fe(CN)_6_]^4−^, 8.44 Å), the amount of ion adsorption for all controlled samples (NaCl-GO, MgCl_2_-GO, and CaCl_2_-GO) shows an uptrend under an increased temperature (Figure 3C), indicating a relatively low energy barrier for adsorption. In the case of the KCl-GO sample (black curve in Figure 3C), which has a quite narrow laminated structure, the adsorption keeps almost constant. This is reasonable as a high energy barrier is required to overcome for the [Fe(CN)_6_]^4−^ ion entering such a small opening at this temperature. 

The impact of interlayer spacing become more apparent in the adsorption of [Fe(CN)_6_]^3−^ ion. As Figure 3D shows, the adsorption amount for [Fe(CN)_6_]^3−^ is not increasing with temperature, except the GO membrane with the largest opening (CaCl_2_-GO, green line). The average opening for the CaCl_2_-GO sample is 13.6 − 3.4 = 10.2 Å, which is bigger than the diameter of hydrated [Fe(CN)_6_]^3−^ (9.50 Å). However, it is worth to note that the remaining Mn^2+^ from the oxidation process [40] could lead to unexpected enlargement of KCl-GO, NaCl-GO and MgCl_2_-GO interlayer spacing, which results in similar increasement of adsorption amount for the larger ion [Fe(CN)_6_]^3−^. 

Apart from using temperature as driving force, we have also studied the adsorption amount of [Fe(CN)_6_]^4−^ and [Fe(CN)_6_]^3−^ with a concentration of 0.1M, 0.3M, 0.5 M and 0.7 M. As shown in Figure 3E,F, the concentration driven adsorption of both ions show similar behaviour to the temperature-driven adsorption. That is, the KCl-GO sample gives the least adsorption of [Fe(CN)_6_]^4−^ ion while the CaCl_2_-GO gives the increasing adsorption amount. The CaCl_2_-GO is the only group of the sample that allows [Fe(CN)_6_]^3−^ to enter in the nanocapillaries within interlayer spacing due to larger opening (10.2 Å). Therefore, it is safe to say that in our experimental conditions (temperature and concentration), the energy barrier cannot be overcome for [Fe(CN)_6_]^4−^ and [Fe(CN)_6_]^3−^ to be adsorbed through a channel with openings smaller than their hydrated size. However, we cannot exclude the possibility of another driving force that allows adsorption of these ions to GO membrane with a well-confined interlayer spacing.

## 4. Conclusions

We have studied the size effect for adsorption in GO membranes with precisely confined interlayer spacing. With suppressed swelling of GO laminates, adsorption of two selected ion species with different sizes has been analyzed with changing temperature and concentration. If the opening is bigger than the hydrated diameter of ions, the adsorption is possible, and its amount increases with temperature and ion concentration. A larger opening of ~10.2 Å provided by interaction of Ca^2+^ allows the sorption of both Fe-cyanide ions but GO modified by smaller ions only allow the smaller Fe-cyanide to penetrate. Neither elevated temperature nor higher concentration could offer the energy required for diffusion of the larger Fe-cyanide ion into the interlayer controlled by smaller ions in the tested range. The observed adsorption of secondary (larger) ions via intercalation of smaller ions through cationic control within the capillaries is a novel phenomenon and requires more detailed research. Our study opens new avenues to understand the adsorption on GO membranes where not only the size of ionic species but also the GO flake size, charge and porosity can play important roles which need further investigations. 

## Data Availability

Data is contained within the article.

## References

[1] Hu M., Mi B. (2014). Layer-by-Layer Assembly of Graphene Oxide Membranes via Electrostatic Interaction. J. Membr. Sci..

[2] Chen L., Shi G., Shen J., Peng B., Zhang B., Wang Y., Bian F., Wang J., Li D., Qian Z. (2017). Ion Sieving in Graphene Oxide Membranes via Cationic Control of Interlayer Spacing. Nature.

[3] Yang J., Hu X., Kong X., Jia P., Ji D., Quan D., Wang L., Wen Q., Lu D., Wu J. (2019). Photo-Induced Ultrafast Active Ion Transport through Graphene Oxide Membranes. Nat. Commun..

[4] Sun P., Zheng F., Zhu M., Song Z., Wang K., Zhong M., Wu D., Little R.B., Xu Z., Zhu H. (2014). Selective Trans-Membrane Transport of Alkali and Alkaline Earth Cations through Graphene Oxide Membranes Based on Cation-π Interactions. ACS Nano.

[5] Zhang X., Wen Q., Wang L., Ding L., Yang J., Ji D., Zhang Y., Jiang L., Guo W. (2019). Asymmetric Electrokinetic Proton Transport through 2D Nanofluidic Heterojunctions. ACS Nano.

[6] Wen Q., Jia P., Cao L., Li J., Quan D., Wang L., Zhang Y., Lu D., Jiang L., Guo W. (2020). Electric-Field-Induced Ionic Sieving at Planar Graphene Oxide Heterojunctions for Miniaturized Water Desalination. Adv. Mater..

[7] Huang H.H., Joshi R.K., De Silva K.K.H., Badam R., Yoshimura M. (2019). Fabrication of Reduced Graphene Oxide Membranes for Water Desalination. J. Membr. Sci..

[8] Cha-Umpong W., Hosseini E., Razmjou A., Zakertabrizi M., Korayem A.H., Chen V. (2020). New Molecular Understanding of Hydrated Ion Trapping Mechanism during Thermally-Driven Desalination by Pervaporation Using GO Membrane. J. Membr. Sci..

[9] Cha-Umpong W., Dong G., Razmjou A., Chen V. (2019). Effect of Oscillating Temperature and Crystallization on Graphene Oxide Composite Pervaporation Membrane for Inland Brine Desalination. J. Membr. Sci..

[10] Sun P., Wang K., Zhu H. (2016). Recent Developments in Graphene-Based Membranes: Structure, Mass-Transport Mechanism and Potential Applications. Adv. Mater..

[11] Jin X., Foller T., Wen X., Ghasemian M.B., Wang F., Zhang M., Bustamante H., Sahajwalla V., Kumar P., Kim H. (2020). Effective Separation of CO2 Using Metal-Incorporated RGO Membranes. Adv. Mater..

[12] Wang L., Wen Q., Jia P., Jia M., Lu D., Sun X., Jiang L. (2019). Light-Driven Active Proton Transport through Photoacid- and Photobase-Doped Janus Graphene Oxide Membranes. Adv. Mater..

[13] Liu Z., Jiang L., Sheng L., Zhou Q., Wei T., Zhang B., Fan Z. (2018). Oxygen Clusters Distributed in Graphene with “Paddy Land” Structure: Ultrahigh Capacitance and Rate Performance for Supercapacitors. Adv. Funct. Mater..

[14] Yang H., Xue T., Li F., Liu W., Song Y. (2019). Graphene: Diversified Flexible 2D Material for Wearable Vital Signs Monitoring. Adv. Mater. Technol..

[15] Zhang Y., Li F., Kong X., Xue T., Liu D., Jia P., Wang L., Ding L., Dong H., Lu D. (2020). Photoinduced Directional Proton Transport through Printed Asymmetric Graphene Oxide Superstructures: A New Driving Mechanism under Full-Area Light Illumination. Adv. Funct. Mater..

[16] Hu G., Xu C., Sun Z., Wang S., Cheng H.M., Li F., Ren W. (2016). 3D Graphene-Foam-Reduced-Graphene-Oxide Hybrid Nested Hierarchical Networks for High-Performance Li-S Batteries. Adv. Mater..

[17] Joshi R.K., Carbone P., Wang F.C., Kravets V.G., Su Y., Grigorieva I.V., Wu H.A., Geim A.K., Nair R.R. (2014). Precise and Ultrafast Molecular Sieving through Graphene Oxide Membranes. Science (New York N. Y.).

[18] Li D., Wang H. (2010). Recent Developments in Reverse Osmosis Desalination Membranes. J. Mater. Chem..

[19] Wan Z., Streed E.W., Lobino M., Wang S., Sang R.T., Cole I.S., Thiel D.V., Li Q. (2018). Laser-Reduced Graphene: Synthesis, Properties, and Applications. Adv. Mater. Technol..

[20] Chu J.Y., Lee K.H., Kim A.R., Yoo D.J. (2019). Graphene-Mediated Organic-Inorganic Composites with Improved Hydroxide Conductivity and Outstanding Alkaline Stability for Anion Exchange Membranes. Compos. Part B: Eng..

[21] Gabunada J.C., Vinothkannan M., Kim D.H., Kim A.R., Yoo D.J. (2019). Magnetite Nanorods Stabilized by Polyaniline/Reduced Graphene Oxide as a Sensing Platform for Selective and Sensitive Non-Enzymatic Hydrogen Peroxide Detection. Electroanalysis.

[22] Chu J.Y., Lee K.H., Kim A.R., Yoo D.J. (2020). Improved Electrochemical Performance of Composite Anion Exchange Membranes for Fuel Cells through Cross Linking of the Polymer Chain with Functionalized Graphene Oxide. J. Membr. Sci..

[23] Wen X., Joshi R. (2020). 2D Materials-Based Metal Matrix. J. Phy. D Appl. Phy..

[24] Wen X., Jin X., Wang F., You Y., Chu D., Zetterlund P.B., Joshi R.K. (2019). Cation-Induced Coagulation in Graphene Oxide Suspensions. Mater. Today Chem..

[25] Zheng S., Tu Q., Urban J.J., Li S., Mi B. (2017). Swelling of Graphene Oxide Membranes in Aqueous Solution: Characterization of Interlayer Spacing and Insight into Water Transport Mechanisms. ACS Nano.

[26] Abraham J., Vasu K.S., Williams C.D., Gopinadhan K., Su Y., Cherian C.T., Dix J., Prestat E., Haigh S.J., Grigorieva I.V. (2017). Tunable Sieving of Ions Using Graphene Oxide Membranes. Nat. Nanotechnol..

[27] Madadrang C.J., Kim H.Y., Gao G., Wang N., Zhu J., Feng H., Gorring M., Kasner M.L., Hou S. (2012). Adsorption Behavior of EDTA-Graphene Oxide for Pb (II) Removal. ACS Appl. Mater. Interfaces.

[28] Chandra V., Kim K.S. (2011). Highly Selective Adsorption of Hg2+ by a Polypyrrole-Reduced Graphene Oxide Composite. Chem. Commun..

[29] Deng J.H., Zhang X.R., Zeng G.M., Gong J.L., Niu Q.Y., Liang J. (2013). Simultaneous Removal of Cd(II) and Ionic Dyes from Aqueous Solution Using Magnetic Graphene Oxide Nanocomposite as an Adsorbent. Chem. Eng. J..

[30] Kotowska U., Kapelewska J., Kotowski A., Pietuszewska E. (2019). Rapid and Sensitive Analysis of Hormones and Other Emerging Contaminants in Groundwater Using Ultrasound-Assisted Emulsification Microextraction with Solidification of Floating Organic Droplet Followed by GC-MS Detection. Water.

[31] Nissinen T.K., Miettinen I.T., Martikainen P.J., Vartiainen T. (2001). Molecular Size Distribution of Natural Organic Matter in Raw and Drinking Waters. Chemosphere.

[32] Cote L.J., Kim J., Tung V.C., Luo J., Kim F., Huang J. (2011). Graphene Oxide as Surfactant Sheets. Pure Appl. Chem..

[33] Lee J., Chae H.R., Won Y.J., Lee K., Lee C.H., Lee H.H., Kim I.C., Lee J. (2013). min Graphene Oxide Nanoplatelets Composite Membrane with Hydrophilic and Antifouling Properties for Wastewater Treatment. J. Membr. Sci..

[34] You Y., Jin X.H., Wen X.Y., Sahajwalla V., Chen V., Bustamante H., Joshi R.K. (2018). Application of Graphene Oxide Membranes for Removal of Natural Organic Matter from Water. Carbon.

[35] Lian B., Deng J., Leslie G., Bustamante H., Sahajwalla V., Nishina Y., Joshi R.K. (2017). Surfactant Modified Graphene Oxide Laminates for Filtration. Carbon.

[36] Klechikov A., Yu J., Thomas D., Sharifi T., Talyzin A.V. (2015). Structure of Graphene Oxide Membranes in Solvents and Solutions. Nanoscale.

[37] Pourbeyram S. (2016). Effective Removal of Heavy Metals from Aqueous Solutions by Graphene Oxide-Zirconium Phosphate (GO-Zr-P) Nanocomposite. Ind. Eng. Chem. Res..

[38] Nightingale E.R. (1959). Phenomenological Theory of Ion Solvation. Effective Radii of Hydrated Ions. J. Phys. Chem..

[39] Mi B. (2014). Graphene Oxide Membranes for Ionic and Molecular Sieving. Science (New York N. Y.).

[40] Yeh C.-N., Raidongia K., Shao J., Yang Q.-H., Huang J. (2015). On the Origin of the Stability of Graphene Oxide Membranes in Water. Nat. Chem..

[41] Shi G., Chen L., Yang Y., Li D., Qian Z., Liang S., Yan L., Li L.H., Wu M., Fang H. (2018). Two-Dimensional Na-Cl Crystals of Unconventional Stoichiometries on Graphene Surface from Dilute Solution at Ambient Conditions. Nat. Chem..

[42] Sun Y.W., Liu W., Hernandez I., Gonzalez J., Rodriguez F., Dunstan D.J., Humphreys C.J. (2019). 3D Strain in 2D Materials: To What Extent Is Monolayer Graphene Graphite?. Phys. Rev. Lett..

